# Monocyte-to-lymphocyte ratio as a predictive marker for breast cancer treatment outcomes: a narrative review

**DOI:** 10.1097/MS9.0000000000003871

**Published:** 2025-09-15

**Authors:** Emmanuel Ifeanyi Obeagu

**Affiliations:** Department of Biomedical and Laboratory Science, Africa University, Mutare, Zimbabwe

**Keywords:** breast cancer, chemotherapy response, immunotherapy, monocyte-to-lymphocyte ratio, prognostic biomarker

## Abstract

Systemic inflammatory markers have gained prominence in cancer research for their prognostic and predictive potential. Among these, the monocyte-to-lymphocyte ratio (MLR) has emerged as a possible indicator of treatment outcomes in breast cancer. This narrative review explores the current evidence regarding MLR as a predictive marker in breast cancer, highlighting its biological rationale, associations with treatment response, and potential clinical relevance. A selective literature search was conducted using PubMed, Scopus, and Web of Science to identify relevant peer-reviewed studies published up to May 2025. Search terms included “monocyte-to-lymphocyte ratio,” “MLR,” “breast cancer,” “prognosis,” and “treatment response.” Articles were selected based on thematic relevance to breast cancer and MLR. Reference lists of key articles were also screened to identify additional sources. No formal quality appraisal tool was applied, as this review does not follow a systematic review framework. The reviewed studies suggest that elevated pre-treatment MLR is associated with poorer outcomes across various breast cancer subtypes and treatment modalities. However, heterogeneity in study design, MLR cut-offs, and patient populations limits direct comparability. MLR represents a promising, readily accessible biomarker with potential applications in breast cancer prognostication and therapy monitoring. Further prospective studies are required to standardize cut-off values and validate clinical utility.

## Introduction

Breast cancer remains the most frequently diagnosed malignancy and the leading cause of cancer-related mortality among women worldwide. Advances in early detection, targeted therapy, and immunotherapy have significantly improved survival rates, yet treatment outcomes remain highly variable. A growing body of research suggests that the tumor microenvironment, particularly systemic inflammation and immune response, plays a critical role in breast cancer progression and treatment efficacy^[1–5]^. Among emerging hematological biomarkers, the MLR has gained increasing attention as a potential predictive and prognostic indicator in breast cancer management^[6,7]^. MLR, calculated as the absolute monocyte count divided by the absolute lymphocyte count, serves as a reflection of systemic immune balance[8]. Monocytes, which differentiate into tumor-associated macrophages (TAMs), contribute to an immunosuppressive and pro-tumorigenic microenvironment, promoting angiogenesis, tumor growth, and metastasis[9]. In contrast, lymphocytes, particularly cytotoxic T cells, play a crucial role in anti-tumor immunity by mounting an immune response against malignant cells. A high MLR suggests a dominance of monocyte-driven inflammation and immune suppression, whereas a lower MLR may indicate a more favorable immune environment for tumor control. Several studies have demonstrated an association between elevated MLR and poor prognosis in breast cancer patients. High MLR values have been linked to lower overall survival (OS) and disease-free survival (DFS), increased recurrence rates, and resistance to conventional treatments. Conversely, patients with lower MLR tend to have better treatment responses and improved survival outcomes. These findings suggest that MLR could serve as a simple yet valuable biomarker for risk stratification and therapeutic decision-making in breast cancer^[10–12]^.

**HIGHLIGHTS**

- **Monocyte-to-lymphocyte ratio (MLR)** reflects immune balance and predicts breast cancer treatment outcomes and prognosis.
- Elevated **MLR is linked to poor survival**, aggressive tumor features, and reduced therapy response.
- **Low pre-treatment MLR** is associated with better response to chemotherapy, especially in neoadjuvant settings.
- MLR is a **simple, low-cost, and widely available** inflammatory biomarker from routine blood tests.
- Integrating MLR with other markers like NLR and PLR enhances **risk stratification and personalized treatment** in breast cancer.

Beyond its prognostic implications, MLR has been increasingly explored as a predictive marker for treatment response. Chemotherapy, a mainstay in breast cancer treatment, elicits varying degrees of response based on the patient’s immune status. Studies indicate that patients with high pre-treatment MLR are more likely to exhibit chemoresistance and have poorer responses to neoadjuvant chemotherapy. In contrast, those with lower MLR often achieve higher rates of pCR, suggesting that MLR could be useful in tailoring chemotherapy strategies^[11,12]^. In the era of immunotherapy, particularly with the use of ICIs, MLR has gained prominence as a potential marker for treatment efficacy. Immunotherapies rely on an intact and responsive immune system to mount an anti-tumor attack, and a low MLR, indicative of higher lymphocyte levels, may correlate with better immunotherapy responses. Preliminary studies suggest that patients with a lower MLR respond more favorably to ICIs, while those with high MLR may exhibit resistance due to immune evasion mechanisms. Integrating MLR with established biomarkers such as PD-L1 expression and TMB could enhance the predictive accuracy of immunotherapy response in breast cancer patients^[13,14]^. MLR may also play a role in predicting responses to targeted therapies, such as endocrine therapy for hormone receptor-positive breast cancer and HER2-targeted treatments. Emerging evidence suggests that an elevated MLR may be associated with resistance to hormone therapy and anti-HER2 agents, necessitating further investigation into its utility in personalized treatment plans. Given the simplicity and accessibility of MLR as a routine blood test, its incorporation into clinical practice could offer a non-invasive and cost-effective tool for guiding treatment decisions^[7,15,16]^.

## Aim

The aim of this review is to explore the prognostic and predictive value of the MLR in breast cancer, with a focus on its role in determining treatment outcomes.

## Review methods

This review adopts a narrative approach to synthesize current knowledge on the predictive role of MLR in breast cancer. A selective literature search was conducted to identify relevant studies that explored associations between MLR and breast cancer treatment outcomes. Searches were performed in PubMed, Scopus, and Web of Science, using combinations of the following keywords: *“monocyte-to-lymphocyte ratio,” “MLR,” “breast cancer,” “treatment response,” “chemotherapy,” “prognostic biomarker,”* and *“inflammation.”* The search included articles published in English up to May 2025. Studies were included based on relevance to the thematic focus of MLR as a predictive or prognostic marker in breast cancer. Additional references were identified through manual screening of bibliographies from key articles. The selection aimed to incorporate diverse study designs (e.g., observational, retrospective, and experimental studies) that provided insights into the clinical relevance of MLR. No formal risk-of-bias assessment or structured appraisal tool was applied, in keeping with the narrative nature of this review. The synthesis of findings was organized around recurring themes including: the biological rationale for MLR as an immune-inflammatory marker, its prognostic and predictive value across breast cancer subtypes, and its implications for clinical practice.

### Limitations

This narrative review is subject to several important limitations that reflect the nature of the existing literature on MLR in breast cancer. Firstly, significant heterogeneity exists across the included studies in terms of study design, patient populations, treatment regimens, and outcome measures. Such variability limits direct comparison and weakens the generalizability of findings. Secondly, the majority of available studies are retrospective in nature, which introduces potential biases related to patient selection, data completeness, and confounding factors that may not be adequately controlled. A further limitation lies in the lack of consensus regarding MLR cut-off values, which varied considerably across studies – from 0.20 to 0.40 in most cases – making it difficult to define standardized thresholds for clinical application. Additionally, there is a potential for publication bias, as studies with significant findings are more likely to be published than those with null results, possibly skewing the perception of MLR’s predictive utility. Finally, there remains a paucity of prospective and randomized controlled trials investigating MLR in breast cancer, which hinders the establishment of causal relationships and limits the robustness of clinical recommendations based on current evidence. Future research should prioritize well-designed, prospective studies with standardized methodologies and clearly defined MLR thresholds to validate its role as a reliable predictive biomarker in breast cancer care.

### Monocyte-to-lymphocyte ratio: a marker of systemic inflammation

Systemic inflammation plays a crucial role in the progression and treatment outcomes of various cancers, including breast cancer. The MLR has emerged as a valuable hematological biomarker reflecting systemic immune status and inflammatory responses. As a simple yet effective parameter derived from routine blood tests, MLR provides insights into the balance between pro-inflammatory monocytes and immune-regulating lymphocytes. An elevated MLR suggests a shift toward a pro-tumorigenic inflammatory environment, which has been associated with poor prognosis and resistance to therapy in cancer patients[16]. Monocytes, a key component of the innate immune system, contribute to cancer progression by differentiating into TAMs. These macrophages support tumor growth through immune suppression, angiogenesis, and extracellular matrix remodeling, creating a microenvironment conducive to metastasis. Conversely, lymphocytes, particularly cytotoxic T cells, play an essential role in anti-tumor immunity by recognizing and eliminating malignant cells. A high MLR, therefore, indicates a dominance of monocyte-driven immune suppression over lymphocyte-mediated tumor defense, which may facilitate cancer cell survival and proliferation^[17,18]^. Given its ability to reflect systemic immune dysfunction, MLR has gained attention as a prognostic and predictive marker in oncology. Studies have linked high MLR values to increased tumor burden, disease progression, and reduced survival rates in breast cancer patients. Additionally, MLR has shown potential in predicting responses to chemotherapy, immunotherapy, and targeted therapy, making it a promising tool for personalized cancer treatment strategies. However, further research is needed to establish standardized cut-off values and validate its clinical utility in diverse patient populations. Understanding the role of MLR in systemic inflammation could help optimize breast cancer management and improve patient outcomes[19].

#### Prognostic value of monocyte-to-lymphocyte ratio in breast cancer

**Figure 1.**
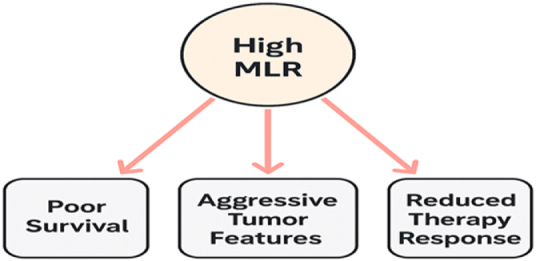
Prognostic value of monocyte-to-lymphocyte ratio in breast cancer.

The MLR has emerged as a significant prognostic biomarker in breast cancer, providing valuable insights into disease progression and patient outcomes. Several studies have demonstrated that an elevated MLR is associated with poor prognosis, including reduced OS and DFS. The prognostic significance of MLR is attributed to its ability to reflect systemic inflammation, which plays a crucial role in tumor progression, immune evasion, and metastasis. A high MLR suggests an imbalance favoring pro-tumorigenic monocytes over anti-tumor lymphocytes, creating an immunosuppressive environment that promotes disease advancement[20]. Clinical studies have consistently shown that breast cancer patients with higher pre-treatment MLR values experience more aggressive disease phenotypes and lower response rates to therapy. For instance, patients with TNBC, a highly aggressive subtype, tend to have elevated MLR, which correlates with increased recurrence rates and reduced survival. Similarly, high MLR levels have been linked to poor outcomes in hormone receptor-positive and HER2-positive breast cancer, indicating its prognostic relevance across different subtypes. The ability of MLR to predict disease severity highlights its potential utility in risk stratification and individualized treatment planning[21]. Despite its prognostic value, challenges remain in establishing standardized MLR cut-off values for clinical application. Variability in patient demographics, tumor biology, and inflammatory conditions may influence MLR levels, necessitating further large-scale studies to validate its use as a routine prognostic marker (Fig. 1). Nevertheless, the simplicity and cost-effectiveness of MLR, derived from routine blood tests, make it a promising tool for oncologists to assess disease progression and tailor treatment approaches. Future research should focus on integrating MLR with other prognostic biomarkers to enhance its predictive accuracy and improve patient outcomes in breast cancer management[22].

#### Monocyte-to-lymphocyte ratio and response to chemotherapy in breast cancer

Chemotherapy remains a cornerstone of breast cancer treatment, particularly for aggressive subtypes such as TNBC and HER2-positive disease. However, response to chemotherapy varies among patients, necessitating reliable biomarkers to predict treatment efficacy. The MLR has emerged as a potential predictor of chemotherapy response, as it reflects the systemic immune balance between pro-tumorigenic monocytes and anti-tumor lymphocytes. Studies suggest that a high pre-treatment MLR is associated with poor chemotherapy response, while a lower MLR may indicate better treatment efficacy and improved patient outcomes. Patients with an elevated MLR tend to exhibit chemoresistance, increased tumor burden, and higher recurrence rates. This is largely due to the immunosuppressive role of monocytes, which differentiate into TAMs and promote an inflammatory microenvironment that facilitates tumor survival. In contrast, lymphocytes, particularly cytotoxic T cells, are crucial for an effective anti-tumor immune response. A lower MLR, indicative of a more active lymphocytic response, has been correlated with higher rates of pCR in patients undergoing neoadjuvant chemotherapy. These findings highlight the importance of systemic immune status in determining chemotherapy outcomes^[12,23–28]^.

#### Monocyte-to-lymphocyte ratio as a predictor of immunotherapy response in breast cancer

Immunotherapy, particularly ICIs, has revolutionized breast cancer treatment, offering new therapeutic options for patients with aggressive and refractory disease. However, not all patients respond favorably to immunotherapy, necessitating reliable biomarkers for predicting treatment efficacy. The MLR has emerged as a promising predictor of immunotherapy response, as it reflects the systemic immune balance between pro-tumorigenic monocytes and anti-tumor lymphocytes. A high MLR, indicative of monocyte-driven immune suppression, has been associated with poor responses to ICIs, whereas a lower MLR, suggesting a more active lymphocytic response, correlates with improved treatment outcomes^[29,30]^. The immunosuppressive effects of monocytes are largely mediated through their differentiation into TAMs, which promote immune evasion by inhibiting T-cell activity and fostering a tumor-permissive microenvironment. High levels of TAMs have been linked to resistance to ICIs, as they reduce the effectiveness of T-cell-mediated anti-tumor responses. Conversely, a lower MLR reflects a more robust lymphocyte-mediated immune response, which is essential for the efficacy of ICIs such as PD-1/PD-L1 inhibitors. Studies suggest that breast cancer patients with a low pre-treatment MLR experience better OS and progression-free survival (PFS) following immunotherapy^[31,32]^.

#### Monocyte-to-lymphocyte ratio and targeted therapies in breast cancer

Targeted therapies have significantly transformed the treatment landscape for breast cancer, particularly in subtypes such as HER2-positive and hormone receptor-positive breast cancers. These therapies, which focus on specific molecular targets involved in cancer cell growth and survival, aim to minimize damage to healthy tissues and improve therapeutic efficacy. However, response to targeted therapies can vary among patients, underscoring the need for reliable biomarkers to predict treatment outcomes. The MLR has shown promise as a potential predictor of response to targeted therapies, as it reflects the systemic immune balance and inflammatory status, both of which influence tumor progression and treatment efficacy[7]. In breast cancer, high MLR has been associated with poor response to targeted therapies, including HER2-targeted treatments such as trastuzumab and hormone therapy for estrogen receptor-positive (ER+) breast cancer. Elevated MLR reflects an increased presence of monocytes, which contribute to a pro-inflammatory and immunosuppressive tumor microenvironment by differentiating into TAMs. These macrophages can promote resistance to targeted therapies by creating an environment that fosters tumor cell survival, angiogenesis, and immune evasion. Conversely, a lower MLR, indicating a more favorable immune profile with a higher proportion of lymphocytes, has been linked to improved therapeutic response and better outcomes following targeted treatment[12]. Furthermore, the prognostic value of MLR in predicting resistance to targeted therapies suggests its potential in guiding personalized treatment plans. For example, in HER2-positive breast cancer, patients with high MLR may be at risk for early disease recurrence or resistance to trastuzumab, while those with lower MLR may experience more favorable treatment outcomes. Similarly, for patients receiving hormone therapy, an elevated MLR may indicate a greater likelihood of therapy resistance and disease progression. Therefore, integrating MLR with other predictive biomarkers, such as HER2 expression or hormone receptor status, could offer a more comprehensive understanding of treatment response and improve therapeutic strategies^[7,12,26]^.

## Conclusion

The monocyte-to-lymphocyte ratio (MLR) has emerged as a promising inflammatory biomarker with potential utility in predicting treatment outcomes in breast cancer. Its appeal lies in its availability, cost-effectiveness, and biological plausibility as a surrogate marker for immune dysregulation within the tumor microenvironment. Several studies have reported associations between elevated pre-treatment MLR and poorer responses to chemotherapy, as well as shorter progression-free and overall survival across various breast cancer subtypes. However, these findings should be interpreted with caution. The available evidence is largely derived from retrospective studies with heterogeneous methodologies, diverse treatment protocols, and inconsistent definitions of high versus low MLR. Moreover, many of the included studies vary in quality, and a standard cut-off value for MLR remains undefined. The absence of large-scale, prospective trials also limits the ability to draw definitive conclusions about its predictive or prognostic value.

While early data suggest that MLR may complement emerging biomarkers such as PD-L1 expression and tumor mutational burden (TMB), these combinatory approaches remain hypothetical and require further exploration in controlled clinical studies. Integration of MLR into multimodal predictive algorithms should be approached cautiously until validated by robust prospective evidence. MLR shows promise as a potential predictor of chemotherapy response and disease progression in breast cancer, but its clinical application is not yet established. Prospective studies, including randomized controlled trials with standardized thresholds and rigorous design, are essential to validate its utility and to understand its role in combination with other immunologic and genomic markers.

## Data Availability

Not applicable as this a narrative review.

## Research registration unique identifying number (UIN)

Not applicable as this a narrative review.
